# Accurate and simple measurement of power generation efficiency and figure of merit of thermoelectric modules based on optical heating and non-contact temperature detection methods

**DOI:** 10.1080/14686996.2025.2551485

**Published:** 2025-08-27

**Authors:** Naoki Nakamura, Fuyuki Ando, Ken-ichi Uchida, Masayuki Murata, Abdulkareem Alasli, Hosei Nagano

**Affiliations:** a Department of Mechanical Systems Engineering, Nagoya University, Nagoya, Japan; b Research Center for Magnetic and Spintronic Materials, National Institute for Materials Science (NIMS), Tsukuba, Japan; c Department of Advanced Materials Science, The University of Tokyo, Kashiwa, Japan; d Research Institute for Energy Efficient Technologies, National Institute of Advanced Industrial Science and Technology (AIST), Tsukuba, Japan

**Keywords:** Thermoelectric figure of merit, thermoelectric generator, laser heating, thermography

## Abstract

In this study, we propose an accurate, simple, and versatile measurement method for power generation efficiency and device figure of merit *ZT* of thermoelectric devices. Toward the energy harvesting applications of thermoelectric generators, the performance characterization under low heat inflow and temperature difference is crucial. However, when the conventional solid-state heat flow meter is used, the uncertainty of power generation performance increases as heat input decreases. We have solved these problems by using a laser for heat input, improving the simplicity and accuracy of power generation efficiency measurements, especially at low heat flow. The direct and non-contact measurement of the temperature difference by using a thermography allowed us to determine *ZT* as well as power generation efficiency. The obtained mean power generation efficiency and *ZT* values are consistent with the values obtained by the conventional method within the error range, thereby validating the reliability of the proposed method. The relative uncertainties of the efficiency and *ZT* were estimated to be less than 3% and 12% for our method, respectively, whereas those were 19% and 24% in situations where the temperature difference was less than 6 K for the conventional method.

## IMPACT STATEMENT

A fully non-contact method was proposed to evaluate *η* and *ZT* of thermoelectric devices, achieving uncertainties of less than 3% and 12%, respectively, using laser heating and thermographic temperature measurement.

## GRAPHICAL ABSTRACT

**Figure:**
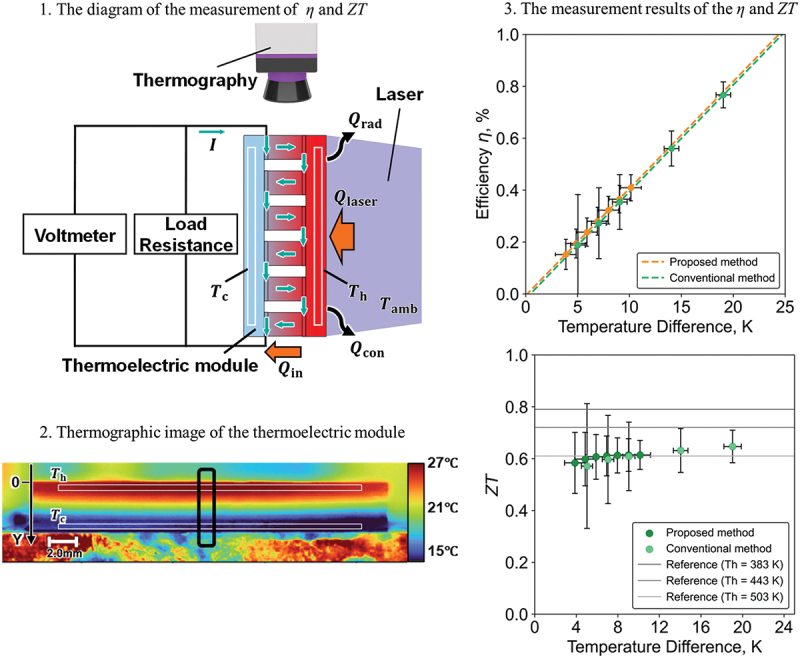

## 1. Introduction

Due to rising energy costs, the depletion of fossil fuel resources and the increasing severity of environmental issues, energy systems are undergoing a significant transition [1]. Thermoelectric generator (TEG) can directly convert heat into electricity and has long been attracting attention as an environmentally friendly energy conversion method [2–5]. TEGs have been widely developed for application in automobiles [6,7], wearable devices [8,9], photovoltaic systems [10–12], and industrial waste heat power generation [13,14] due to their advantages of frictionless operation, high reliability and stability, and low installation and maintenance costs [15–18]. In recent years, a combined Internet of Things (IoT)-thermoelectric system has also been considered, in which thermoelectric devices are used to generate electricity from factory waste heat to provide an independent power source for IoT devices. As TEGs are operated and systematized in these various fields, it is becoming more and more important to ensure stable power output. In particular, accurate performance evaluation at low heat input is increasingly required in the field of energy harvesting [19,20].

The performance of thermoelectric devices is typically characterized in terms of the following two important parameters. One is the power generation efficiency *η*, which indicates how much output power is obtained from the input heat flow. The other is the device thermoelectric figure of merit *ZT*, which determines the thermoelectric performance of the device itself [21,22]. Proper evaluation of *η* requires simultaneous measurement of input heat flow $Q_{\mathrm{in}}$ and output power $P_{\mathrm{out}}$ according to the following equation:

(1) $$\eta =\frac{P_{\mathrm{out}}}{Q_{\mathrm{in}}}$$

The conventional method to determine $Q_{\mathrm{in}}$ is a steady-state heat flow method using a solid-state heat flow meter (HFM) [23–26]. In this method, $Q_{\mathrm{in}}$ can be calculated using the 1D Fourier’s law from the temperature difference $\Delta T_{M}$ within HFM. $P_{\mathrm{out}}$ is simultaneously measured by a four terminal method, followed by the calculation of *η*. However, this method has several limitations. Firstly, we need to arrange the area of HFM with that of the thermoelectric device to realize the one-dimensional heat transport. Secondly, as $\Delta T_{M}$ within HFM becomes smaller, the uncertainty of the absolute temperature causes a larger error in the estimated $Q_{\mathrm{in}}$. Meanwhile, *ZT* is mainly used to compare the thermoelectric performance of various devices without the effect of Carnot efficiency as follows:

(2) $$\mathrm{ZT}={\left(\frac{T_{h}-T_{c}\left(1-\eta_{\max}\right)}{T_{h}\left(1-\eta_{\max}\right)-T_{c}}\right)}^{2}-1$$

Although *ZT* is the common indicator in the field of thermoelectrics, the accurate measurement especially at low $Q_{\mathrm{in}}$ has been difficult mainly due to the uncertainty of $\Delta T_{M}$ within HFM and hence $Q_{\mathrm{in}}$.

This study presents a new method to characterize thermoelectric performance using a laser and a thermography. Because we directly estimate $Q_{\mathrm{in}}$ from an injected laser power [27–32] instead of a solid-state heat flow sensor, the measurement error is significantly reduced even in the low $Q_{\mathrm{in}}$ condition. This enables an accurate measurement of *η* over a wide range of $Q_{\mathrm{in}}$, which has been difficult for the conventional method. In addition, the area of the laser injection can be flexibly changed according to the thermoelectric modules with various sizes and shapes. Furthermore, the proposed method can simultaneously and precisely measure *ZT* in low heat input by directly measuring the temperature distribution in the module. We applied this method to a commercially available thermoelectric device with the known *η* and *ZT* and compared the results with those obtained by the conventional method using HFM. As a result, our data shows a quantitative agreement with a specification of the device [33] and much lower error in low heat input than that by the conventional method. The proposed method will contribute to the acceleration of energy harvesting applications of thermoelectric devices.

## 2. Measurement principles and setup

### 2.1. Measurement principles

**Figure 1.**
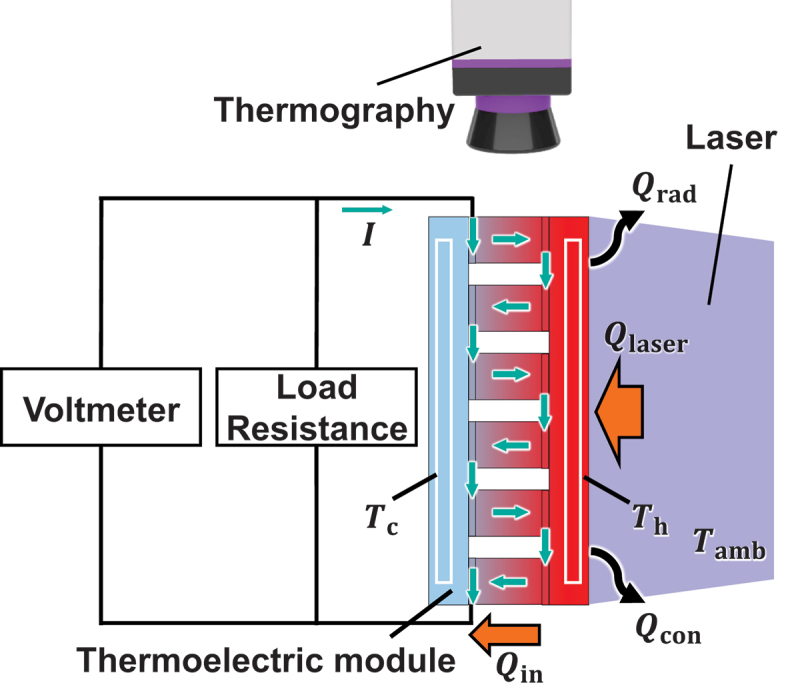
The diagram of the simultaneous measurement of the power generation efficiency *η* and device figure of merit *ZT*.

Figure 1 shows the schematic of our measurement principle. $Q_{\mathrm{in}}$ is estimated by measuring the heat absorbed from the laser on the heating surface and subtracting the heat loss from the surface. The relationship between $Q_{\mathrm{in}}$ and the laser output $Q_{\mathrm{laser}}$ can be described by Equation (3), which incorporates the absorptance α (using black coating) of the device surface and the convective heat loss $Q_{\mathrm{con}}$ and radiative heat loss $Q_{\mathrm{rad}}$ to the surroundings:

(3) $$Q_{\mathrm{in}}=\alpha Q_{\mathrm{laser}}-Q_{\mathrm{con}}-Q_{\mathrm{rad}}$$

$Q_{\mathrm{con}}$ and $Q_{\mathrm{rad}}$ are given by

(4) $$Q_{\mathrm{con}}=\mathrm{hA}\left(T_{h}-T_{\mathrm{amb}}\right)$$

(5) $$Q_{\mathrm{rad}}=\mathrm{\varepsilon \sigma A}\left(T_{h}^{4}-T_{amb}^{4}\right)$$

respectively, where $T_{\mathrm{amb}}$ is the ambient temperature, h=10 the heat transfer coefficient between the device surface and air, *A* the area of the device surface, and *ε* the emissivity of the surface. $P_{\mathrm{out}}$ is measured from Equation (6) by attaching a variable load resistance $R_{\mathrm{out}}$:

(6) $$P_{\mathrm{out}}=\mathrm{IV}=\frac{V^{2}}{R_{\mathrm{in}}+R_{\mathrm{out}}}$$

where *I*, *V*, and $R_{\mathrm{in}}$ are the current, voltage, and internal resistance of the thermoelectric device, respectively. The value of $R_{\mathrm{out}}$ is varied, and the maximum output power $P_{\max}$ is calculated from the load resistance dependence. Then, since the value of $Q_{\mathrm{in}}$ is a constant value, we can calculate the maximum efficiency from $Q_{\mathrm{in}}$ and $P_{\max}$.

(7) $$\eta_{\max}=\frac{P_{\max}}{Q_{\mathrm{in}}}$$

*ZT* can be derived from the one-dimensional heat conduction equation and the electric circuit theory, and the equation is expressed as in Equation (7) using the value of maximum power generation efficiency $\eta_{\max}$ and $T_{h}$ and $T_{c}$ [24]

### 2.2. Measurement apparatus

**Figure 2.**
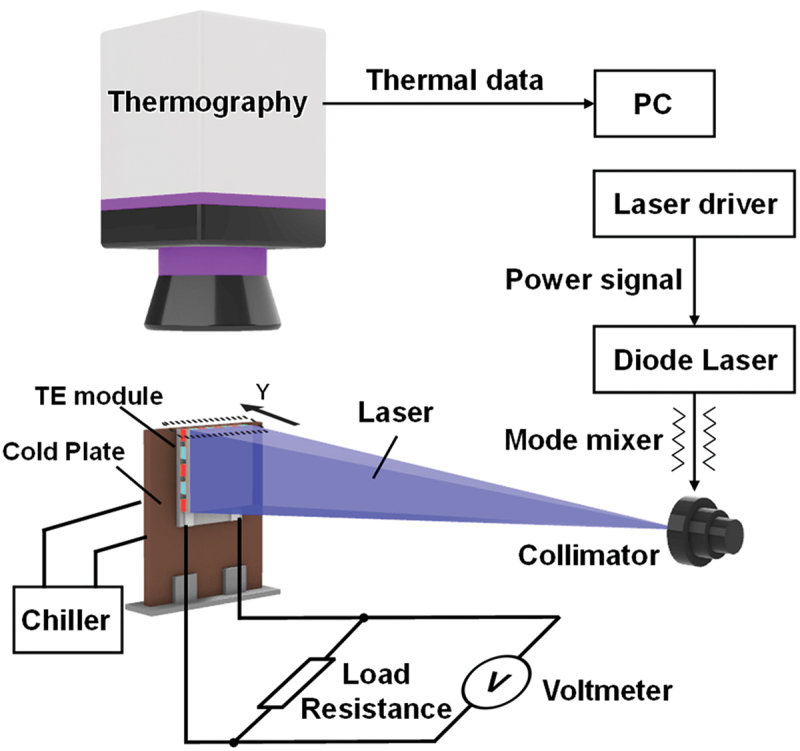
Schematic diagram of the measurement apparatus.

Figure 2 shows a schematic diagram of the apparatus for this measurement. The apparatus consists mainly of a diode laser, thermography, variable resistor, voltmeter, chiller, cold plate, and PC. The diode laser with a wavelength of 455 nm was used for the thermal input to the thermoelectric device. The laser intensity value was set to be the actual intensity output from the laser as measured with a power meter, and the input shape of the laser was adjusted to match the shape of the module. A mode mixer was also used to ensure uniform heating within the surface. $T_{h}$ and $T_{c}$ were determined by observing the side surface of the device with the thermography, sending the data to the PC, and then averaging the temperatures in the areas of aluminum oxide plates for the hot and cold sides, respectively. The cold side of the device was bonded to the cold plate, and the temperature on the cold side was controlled by the chiller. Then, the laser intensity to heat the module surface was set by the PC, and heat input was started. The laser-heated surfaces of the module and the observation surfaces of the thermography were coated with black paint (JAPAN SENSOR JSC-3). In this measurement, the optical absorbance at the laser wavelength (455 nm) and the emissivity near room temperature (corresponding to wavelengths around 10 µm) were set to 0.983 and 0.94, respectively, using the experimentally measured value obtained with the UV–Vis spectrophotometer (V-670, JASCO) and the catalog value [34]. After $T_{h}$ and $T_{c}$ were stabilized within ±0.1°C, the value of the load resistance was varied to find the maximum $P_{\mathrm{out}}$. $Q_{\mathrm{in}}$ was calculated from Equations (3)-(5) by substituting the values of $T_{h}$ and $T_{c}$. By repeating this process with different laser intensities, the *η* and *ZT* values at various temperature differences $\Delta T=T_{h}-T_{c}$ were calculated. This thermography is calibrated using a black plate with the emissivity of 0.94 before each measurement [35]. In addition, the temperature measurement is calibrated periodically by inter-measurement with a thermocouple coated with black paint.

## 3. Measurement

### 3.1. Verification sample and measurements conditions

The module used in this study is the SINGLE-STAGE THERMOELECTRIC GENERATOR TG12–2.5, and its specifications are summarized in Table 1 [33]. It should be noted that the *ZT* value varies depending on the measured temperature range: 0.61, 0.72 and 0.79 at $T_{h}=110,170,\mathrm{and}\;\;230$°C, respectively, with the fixed $T_{c}=50$°C, indicating the large temperature dependence of the thermoelectric properties for the constituent thermoelectric legs.

**Table 1.** Specifications of sample module [33]. The load resistance for optimum *η* and *ZT* values are measured in $T_{h}=110,170,230$°C and $T_{c}=50$°C.

Ceramic Area	Module Hight	AC Resistance	Load Resistance for Optimum *η*	*ZT*
30 mm × 30 mm	3.94 mm	4.47–5.69 Ω	10.47, 9.68, 8.75 Ω	0.61, 0.72, 0.79

The experimental conditions were as follows: the cold plate temperature was set at 15°C and the laser power was varied from 1500 mW to 4500 mW in increments of 500 mW. Measurements were conducted in the atmosphere. An average value was taken from five measurements at each laser power setting.

### 3.2. Temperature measurements

**Figure 3.**
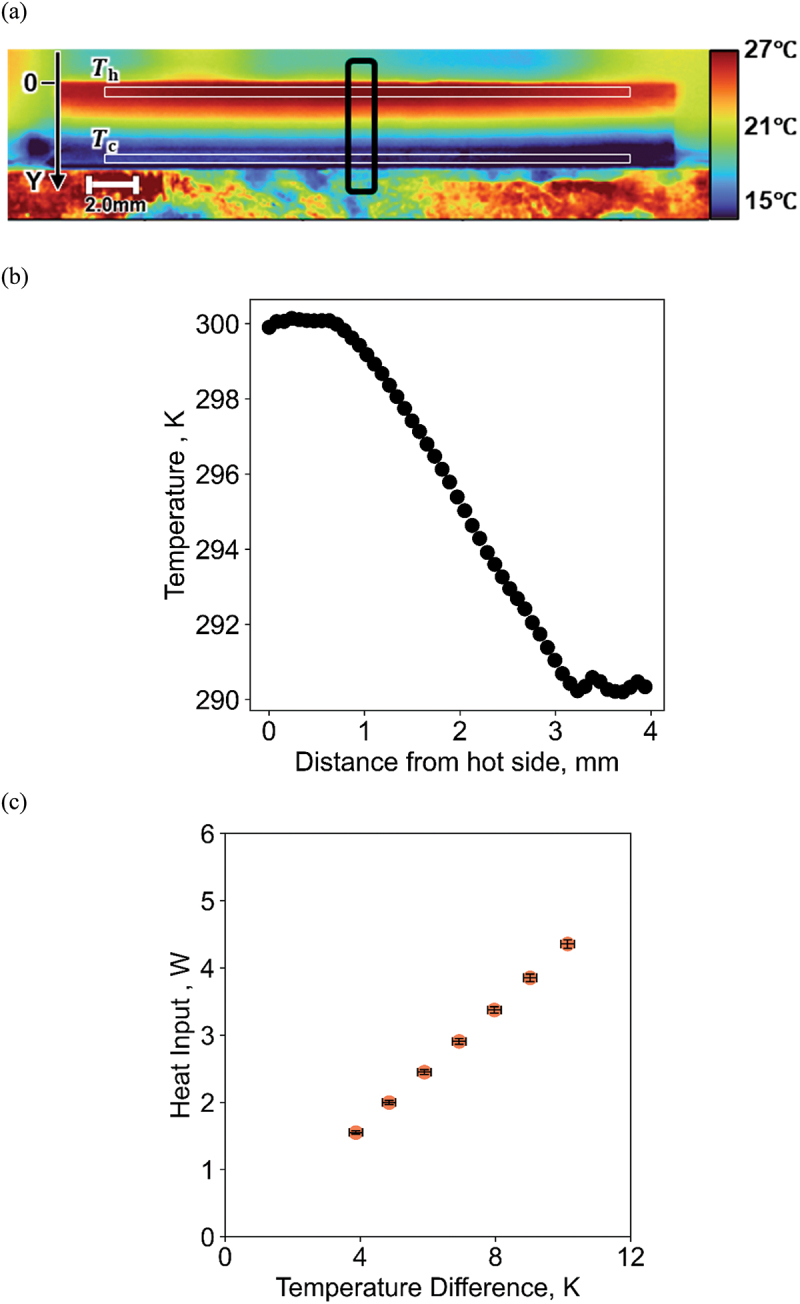
(a) Thermographic image of the side surface of the thermoelectric module when 4500 mW is applied, (b) line scan data in the direction of heat flow in the black area marked in (a), (c) $Q_{\mathrm{in}}$ as a function of T.

Figure 3(a) shows an example of the thermography image data at the laser power of 4500 mW, where $T_{h}$ and $T_{c}$ are defined around the central area of aluminum oxide plates to eliminate the influence of heat loss near the edges. The standard deviation of the temperature was less than 0.4°C in all measurement conditions. Figure 3(b) showed the averaged line scan data in the direction of heat flow in the black area marked in Figure 3(a). Figure 3(c) shows ΔT dependence of $Q_{\mathrm{in}}$ calculated using the above data. As shown in Figure 3(a, b), the surface was homogeneously heated realizing a one-dimensional heat transport in Y-direction.

### 3.3. Output power measurement

**Figure 4.**
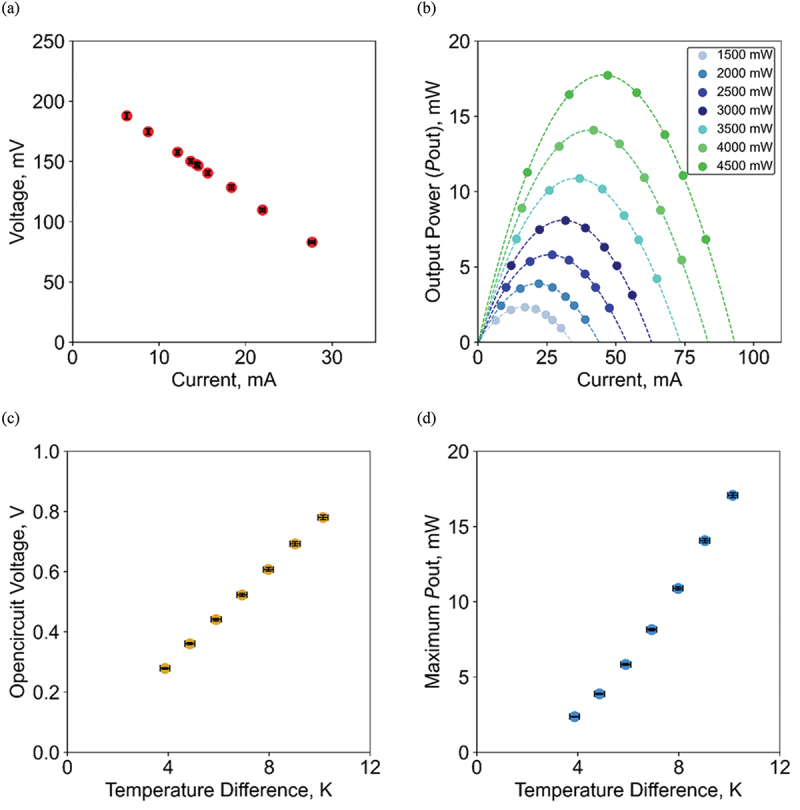
Thermoelectric generation properties obtained by the proposal method. (a) *I-V* diagram at 1500 mW laser power, (b) the output power for the current at each laser power condition, (c) the open-circuit voltage at each temperature difference, and (d) maximum $P_{\mathrm{out}}$.

We characterized the maximum $P_{\mathrm{out}}$ with varying $R_{\mathrm{out}}$ under the application of $Q_{\mathrm{in}}$. Firstly, the DC internal resistance of the thermoelectric device $R_{\mathrm{in}}$ at each condition was calculated from the slope of the measured *I-V* curve, where *I* is tuned by changing $R_{\mathrm{out}}$. The $R_{\mathrm{in}}$value calculated from Figure 4(a) is 4.81 Ω, which is consistent with the catalog reference value in Table 1. Figure 4(b) shows the *I* dependence of $P_{\mathrm{out}}\left(=\mathrm{IV}\right)$ at various $Q_{\mathrm{in}}$ values, where $R_{\mathrm{out}}$ for the maximum output condition at 1500 mW was 8.52 Ω, which is also confirmed to be close to the reference value of optimum *η*. Based on these data, Figure 4(c, d) show the open-circuit voltage and maximum $P_{\mathrm{out}}$ versus ΔT at each laser output. Here, since all the measurements were performed around room temperature, the open-circuit voltage is proportional to ΔT, and the maximum $P_{\mathrm{out}}$ increases in proportion to the square of ΔT with neglecting the temperature dependence of thermoelectric properties of the constituent thermoelectric legs.

## 4. Error analysis and experimental results

### 4.1. Error analysis

We describe the error analysis procedure in detail. The relative uncertainty of $Q_{\mathrm{in}}$ takes into account the uncertainty in $Q_{\mathrm{laser}}$, α, A, $T_{h}$ and variations in the heat transfer coefficient *h*, which ranges from 7 to 15 W/m^2^ K under natural air-cooled environment. Meanwhile, the relative uncertainty of the $P_{\mathrm{out}}$ includes the variable resistance value and the uncertainty in the *V* measurement. From the law of error propagation, the relative uncertainties are, respectively, expressed by the following equations:

(8) $$\frac{u_{\mathrm{proposed}}\left(Q_{\mathrm{in}}\right)}{Q_{\mathrm{in}}}=\sqrt{\begin{array}{c} {\left(\frac{\partial Q_{\mathrm{in}}}{\partial \alpha }\delta \alpha \right)}^{2}+{\left(\frac{\partial Q_{\mathrm{in}}}{\partial Q_{\mathrm{laser}}}\delta Q_{\mathrm{laser}}\right)}^{2}+{\left(\frac{\partial Q_{\mathrm{in}}}{\partial h}\mathrm{\delta h}\right)}^{2}+{\left(\frac{\partial Q_{\mathrm{in}}}{\partial A}\mathrm{\delta A}\right)}^{2}\\ +{\left(\frac{\partial Q_{\mathrm{in}}}{\partial \varepsilon }\delta \varepsilon \right)}^{2}+{\left(\frac{\partial Q_{\mathrm{in}}}{\partial T_{h}}\delta T_{h}\right)}^{2}+{\left(\frac{\partial Q_{\mathrm{in}}}{\partial T_{\mathrm{amb}}}\delta T_{\mathrm{amb}}\right)}^{2} \end{array}}$$

(9) $$\frac{u_{\mathrm{proposed}}\left(P_{\mathrm{out}}\right)}{P_{\mathrm{out}}}=\sqrt{{\left(\frac{\partial P_{\mathrm{out}}}{\partial R_{\mathrm{out}}}\delta R_{\mathrm{out}}\right)}^{2}+{\left(\frac{\partial P_{\mathrm{out}}}{\partial V}\mathrm{\delta V}\right)}^{2}}$$

Based on these equations, the relative uncertainty of *η* is given by

(10) $$\frac{u_{\mathrm{proposed}}\left(\eta \right)}{\eta }=\sqrt{{\left(\frac{\partial \eta }{\partial Q_{\mathrm{in}}}\delta Q_{\mathrm{in}}\right)}^{2}+{\left(\frac{\partial \eta }{\partial P_{\mathrm{out}}}\delta P_{\mathrm{out}}\right)}^{2}}$$

In the steady-state heat flow method using HFM (hereinafter, referred to as the conventional method) [36], $Q_{\mathrm{in}}$ is expressed as

(11) $$Q_{\mathrm{in}}=Q_{\mathrm{out}}+P_{\mathrm{out}}$$

(12) $$Q_{\mathrm{out}}=\mathrm{kA}\left(T_{u}-T_{b}\right)$$

where $Q_{\mathrm{out}}$ represents the heat flow from the cold side of the module, *k* is the thermal conductivity of HFM, and $T_{u}$, $T_{b}$ are the temperatures at the hot and cold side temperatures in HFM. Therefore, the relative uncertainty of *η* for the conventional method is expressed as:

(13) $$\frac{u_{\mathrm{conventional}}\left(Q_{\mathrm{in}}\right)}{Q_{\mathrm{in}}}=\sqrt{\begin{array}{c} {\left(\frac{\partial Q_{\mathrm{in}}}{\partial k}\mathrm{\delta k}\right)}^{2}+{\left(\frac{\partial Q_{\mathrm{in}}}{\partial A}\mathrm{\delta A}\right)}^{2}+{\left(\frac{\partial Q_{\mathrm{in}}}{\partial T_{u}}\delta T_{u}\right)}^{2}\\ +{\left(\frac{\partial Q_{\mathrm{in}}}{\partial T_{b}}\delta T_{b}\right)}^{2}+{\left(\frac{\partial Q_{\mathrm{in}}}{\partial P_{\mathrm{out}}}\delta P_{\mathrm{out}}\right)}^{2} \end{array}}$$

(14) $$\frac{u_{\mathrm{conventional}}\left(P_{\mathrm{out}}\right)}{P_{\mathrm{out}}}=\sqrt{{\left(\frac{\partial P_{\mathrm{out}}}{\partial R_{\mathrm{out}}}\delta R_{\mathrm{out}}\right)}^{2}+{\left(\frac{\partial P_{\mathrm{out}}}{\partial V}\mathrm{\delta V}\right)}^{2}}$$

It should be noted that it is assumed that the uncertainty due to $P_{\mathrm{out}}$ is negligibly small in the conventional method. The relative uncertainty of *ZT* is calculated from the following equation:

(15) $$\frac{u\left(\mathrm{ZT}\right)}{\mathrm{ZT}}=\sqrt{{\left(\frac{\partial \mathrm{ZT}}{\partial T_{h}}\delta T_{h}\right)}^{2}+{\left(\frac{\partial \mathrm{ZT}}{\partial T_{c}}\delta T_{c}\right)}^{2}+{\left(\frac{\partial \mathrm{ZT}}{\partial \eta }\delta \eta \right)}^{2}}$$

ΔT used as the horizontal axis when graphing *η* and *ZT* includes the measurement error of each temperature measurement method. The uncertainty of each parameter together with the temperature measurement error for each temperature measurement method is summarized in Table 2.

**Table 2.** Uncertainty budget of the evaluation of each parameter at laser power of 1500 mW.

Uncertainty components		Relative standard uncertainty
Proposed method efficiency *u*(*η*)/*η*		3.0%
Proposed method figure of merit *u*(*ZT*)/*ZT*		11.4%
Heat flow measurement	Heat transfer coefficient, W/m^2^/K	3.4%
	Emissivity of the surface	1.0%
	Laser absorption	1.0%
	Module area, m^2^	1.0%
	Laser intensity, W	1.0%
Temperature measurement	Thermography, K (proposed)	1.0°C
	Thermocouple, K (conventional)	0.5°C
	Atmosphere, K (proposed)	1.0°C
Electric power measurement	Electrical resistance, Ω	1.0%
	Voltage measurement, V	1.0%

### 4.2. Experimental results and discussion

**Figure 5.**
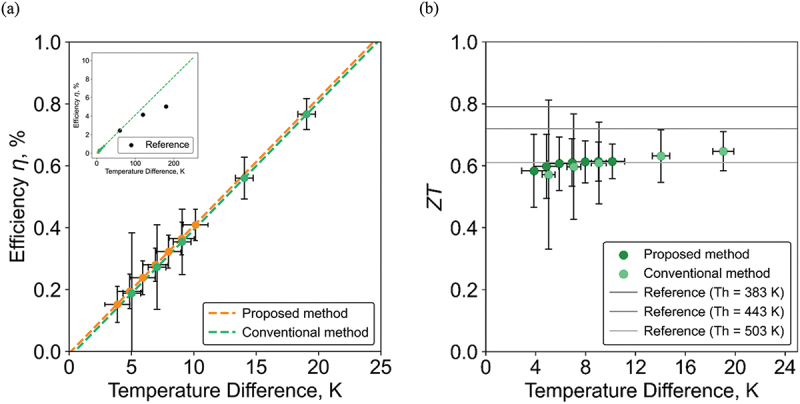
Comparison of the proposed method with the conventional method. (a) The power generation efficiency and (b) *ZT* value as a function of the temperature difference.

To confirm the validity of the proposed method, we performed a comparative measurement of *η* and *ZT* using the conventional method using HFM developed by the National Institute of Advanced Industrial Science and Technology (AIST) [36]. The measurement conditions were adjusted to maintain $T_{c}$ at 15°C for both the proposed and the conventional methods. Figure 5(a) shows that the *η* values of the same device obtained by the proposed and conventional methods. Due to the effect of Carnot efficiency, *η* monotonically increases as ΔT increases. Importantly, the *η* values clearly match between these methods within a margin of error. As a possible reason for the slight difference between these methods, it should be noted that the conventional method uses the temperatures of the heat source and sink for $T_{h}$ and $T_{c}$, which may cause an estimation of larger ΔT in the conventional method by including contact thermal resistances. The measurement uncertainty of the proposed method is within 3% at ΔT=4K whereas that of the conventional method is 19% at ΔT=5K, ensuring the higher reliability of our method even in the low $Q_{\mathrm{in}}$ condition.

Figure 5(b) shows the comparison of the *ZT* values between the proposed and conventional methods and also the catalog values of the same device. As a result of Equation (2) excluding the contribution of the Carnot efficiency from *η*, the *ZT* values are almost constant regardless of ΔT in our measurement range. The *ZT* values for the two methods agree with each other within the error bar. In our method, the standard deviation is less than 2% and the relative error is less than 12% even at low heat flow input, whereas the relative error for the conventional method is about 24%. These results indicate that the proposed method is promising for accurate *ZT* measurements with small temperature differences, which was difficult to achieve with the conventional method.

## 5. Conclusion

We have developed a simple and accurate measurement method using a laser and thermography to simultaneously evaluate the power generation efficiency *η* and the device figure of merit *ZT* of thermoelectric devices. The *η* and *ZT* values estimated from the proposed and conventional methods agree with each other within the margin of error. The error analysis reveals that the proposed method shows higher accuracy than the conventional method; the results for the proposed method show an uncertainty of less than 3% for *η* and less than 12% for *ZT* with the small temperature difference of 4 K. The proposed method has the versatility to evaluate thermoelectric devices with various dimensions, providing a simpler and more adaptable measurement method. By reducing the complexity of the evaluation process of thermoelectric devices, this method will significantly contribute to the design and optimization of thermoelectric applications including energy harvesting, where efficiency measurements at small temperature gradients are essential.

## Data Availability

The data that support the findings of this study are available from the corresponding authors upon reasonable request.
